# Correlates of Walking for Transportation and Use of Public Transportation Among Adults in St Louis, Missouri, 2012

**DOI:** 10.5888/pcd11.140125

**Published:** 2014-07-03

**Authors:** Marissa L. Zwald, James A. Hipp, Marui W. Corseuil, Elizabeth A. Dodson

**Affiliations:** Author Affiliations: James A. Hipp, Elizabeth A. Dodson, Brown School, Washington University in St Louis, St Louis, Missouri; Marui W. Corseuil, Federal University of Santa Catarina, Santa Catarina, Brazil.

## Abstract

**Introduction:**

Attributes of the built environment can influence active transportation, including use of public transportation. However, the relationship between perceptions of the built environment and use of public transportation deserves further attention. The objectives of this study were 1) to assess the relationship between personal characteristics and public transportation use with meeting national recommendations for moderate physical activity through walking for transportation and 2) to examine associations between personal and perceived environmental factors and frequency of public transportation use.

**Methods:**

In 2012, we administered a mail-based survey to 772 adults in St Louis, Missouri, to assess perceptions of the built environment, physical activity, and transportation behaviors. The abbreviated International Physical Activity Questionnaire was used to assess walking for transportation and use of public transportation. The Neighborhood Environment Walkability Scale was used to examine perceptions of the built environment. Associations were assessed by using multinomial logistic regression.

**Results:**

People who used public transportation at least once in the previous week were more likely to meet moderate physical activity recommendations by walking for transportation. Age and employment were significantly associated with public transportation use. Perceptions of high traffic speed and high crime were negatively associated with public transportation use.

**Conclusion:**

Our results were consistent with previous research suggesting that public transportation use is related to walking for transportation. More importantly, our study suggests that perceptions of traffic speed and crime are related to frequency of public transportation use. Future interventions to encourage public transportation use should consider policy and planning decisions that reduce traffic speed and improve safety.

## Introduction

Regular physical activity is essential to overall health. It is associated with weight control; reduced risk of many chronic diseases such as coronary heart disease, stroke, diabetes, hypertension, colon cancer, breast cancer, and depression; and the prevention of early death (1,2). Despite the well-established health benefits of regular physical activity, fewer than half of US adults meet the recommended levels of physical activity outlined in the 2008 Physical Activity Guidelines for Americans, to engage in at least 150 minutes of moderate-intensity aerobic activity each week (1,3).

Walking for transportation, which can include walking at the beginning or end of a trip taken by public transportation, can provide opportunities for meeting recommended levels of physical activity. Previous studies have demonstrated that people who walk to and from public transportation stops engage in more daily physical activity than those who do not (4–9). Rissel and colleagues (10) recently reviewed 9 public transportation and physical activity studies and found that although these studies used different measures of physical activity, public transportation use was associated with at least 8 additional minutes of daily physical activity, with some studies reporting a range of 12 to 15 additional minutes (10). More evidence is needed, however, to understand the association between public transportation use and physical activity and, more specifically, the built environment features that may influence this relationship (8).

The association between walking for transportation and objectively measured attributes of the built environment, such as residential density, land use mix, street connectivity, and accessibility to destinations, is well-documented (11–15). A recent study by Wasfi and colleagues (16) examined the relationship between objectively measured characteristics of the built environment, including residential density, land use mix, and street connectivity, and walking to public transportation; contrary to the aforementioned studies (11–15), the investigators found no significant associations. Moreover, a growing body of evidence has also suggested that perceptions of built environment characteristics can influence transportation-related physical activity, including walking for transportation. Some of these perceived environmental factors include perceived personal safety from crime and traffic, accessibility to walking and bicycling facilities, aesthetics, residential density, land use mix, and street connectivity (11,15,17–19). However, little is known about how perceptions of the built environment influence public transportation use. Although communities that support walking for transportation tend to also encourage public transportation use (8), understanding the perceived environmental factors specifically related to public transportation use could inform improved policy and planning decisions on the built environment and public transit systems.

Our study had 2 objectives. The first was to further assess the relationship between personal characteristics and use of public transportation and walking for transportation. The second objective was to examine associations between personal and perceived environmental factors with frequency of public transportation use.

## Methods

### Study setting

This study was conducted in St. Louis, Missouri, from July through December 2012. In 2012, St. Louis had an area of 62 square miles and a population of 318,172, of which approximately 52% were female, 79% were aged 18 years or older, 16% had a total household income less than $10,000 per year, and 56% were employed outside of the home (20). The public transportation system in St. Louis is operated by Metro St. Louis, which at the time of data collection offered bus services with 75 different routes, a light rail train system serving 37 stations in the greater St. Louis area, and call-a-ride shuttles for people with disabilities. Metro St. Louis offered reduced-fare programs for seniors (≥65 y) and persons with disabilities (21).

Our study was part of a broader investigation of neighborhood conditions, physical activity, transportation behaviors, and personal characteristics that was approved by the institutional review board of Washington University in St. Louis. We used simple random sampling to administer a mail-based survey to 2,898 households in St. Louis (refers throughout this article to the city of St. Louis only and not to St. Louis County). An adult aged 18 years or older who resided at the address to which the survey was mailed was instructed to complete the survey, and only 1 survey per household was considered for the study. A total of 772 surveys were collected, an overall response rate of 26.6%.

### Measures

#### Dependent variables

The International Physical Activity Questionnaire (IPAQ), long-form, was used to measure walking for transportation and public transportation use in the past 7 days. Walking for transportation was categorized according to the most recent United States Physical Activity Guidelines for duration of moderate physical activity (22). The 3 response categories for this variable were 1) met national guidelines for moderate physical activity by walking for transportation (≥150 min/wk); 2) engaged in some walking for transportation but did not meet national guidelines (1–149 min/wk); or 3) engaged in no walking for transportation (0 min/wk) (1). To understand differences across regular transit users, occasional transit users, and transit nonusers, the number of days traveled by bus or train in the previous week was categorized as 5 or more days per week, 1 to 4 days per week, or 0 days per week. The International Physical Activity Questionnaire has been previously validated (22).

#### Independent variables

The following individual factors were included in the analysis: sex, age (18–29 y, 30–39 y, 40–49 y, 50–59 y, ≥60 y), annual household income level (<$10,000, $10,000–$29,999, $30,000–$49,999, $50,000–$69,999, ≥$70,000), and employment status (employed or unemployed).

To assess perceptions of built environment characteristics, items from the abbreviated version of the Neighborhood Environment Walkability Scale (NEWS) were selected by analyzing previous research and by including variables hypothesized to influence public transportation use (11–19,23). A previous study conducted by Adams et al. demonstrated good concurrent validity for self-reported built environment items included in NEWS with objective Geographic Information System measures (24). For the current study, 4 items were used to assess perceptions of traffic safety: 1) traffic volume, 2) traffic speed, 3) presence of crosswalks and pedestrian signals, and 4) visibility of pedestrians and bicyclists. Four items were used to measure perceptions of personal safety: 1) perceived nearby crime, 2) safety from crime when walking during the day, 3) safety from crime when walking at night, and 4) street lighting at night. One item was used to assess perceptions of accessibility to sidewalks. These 9 NEWS items were measured by using 4-point Likert-scale–type questions (strongly agree to strongly disagree). The response categories were dichotomized (agree vs disagree).

Perceived accessibility to neighborhood destinations was assessed by using NEWS items that asked participants to report the number of minutes required to walk from the participant’s home to the nearest of 20 specific businesses or facilities, such as a supermarket, post office, or coffee shop. The 20 items were collapsed into a single destinations measure representing the number of destinations within a 10-minute walk. Responses were categorized as 0 destination, 1 to 5 destinations, 6 to 10 destinations, and 11 or more destinations.

#### Statistical analysis

Descriptive statistics and frequencies were used to describe individual participant characteristics, frequency of participants meeting national recommendations for moderate physical activity by walking for transportation, and frequency of public transportation use. Bivariate associations between each personal characteristic and public transportation use with the dependent variable of walking for transportation were evaluated by using multinomial logistic regression to estimate the odds ratios (ORs) and 95% confidence intervals (CIs). To create more parsimonious models, only those characteristics with a value of *P* ≤ .20 by Wald’s test in the bivariate associations were included in the final analysis examining the associations between categories of minutes spent walking for transportation (25). The reference category was respondents who reported not walking for transportation in the previous week. *P* ≤.05 was considered statistically significant in the final model.

To assess correlates of public transportation use, the analysis steps described above were used, including personal and perceived built environment characteristics as independent variables and frequency of public transportation use as the dependent variable. The reference category was transit nonuser. All analyses were performed using Stata version 12.0 (STATA Corp, College Station, Texas).

## Results

### Description of the sample

Compared with the demographic composition of St. Louis at the time of our study (20), our sample had a higher proportion of female participants (64%) and a higher percentage of respondents who earned less than $10,000 per year (27%), but our sample was similarly represented across other individual characteristics assessed (Table 1). The majority of the sample (57%) was employed outside the home, and most participants were younger than 50 years (58%).

**Table 1:** Participant (N = 772) Characteristics, Survey of Transportation Use, St. Louis, Missouri, 2012

Characteristic	% (n)^a^
**Sex**	
Male	36.4 (275)
Female	63.6 (480)
**Age, y**	
18–29	16.8 (127)
30–39	22.6 (171)
40–49	18.6 (141)
50–59	19.1 (145)
≥60 or over	23.0 (174)
**Income, $**	
<10,000	27.0 (202)
10,000–29,999	24.3 (182)
30,000–49,000	16.4 (123)
50,000–69,000	11.6 (87)
≥70,000	20.6 (154)
**Employment status**	
Unemployed	42.9 (331)
Employed	57.1 (440)
**Minutes walked in preceding week**	
0	34.3 (263)
1–149	34.7 (266)
≥150	31.0 (238)
**No. days used public transportation in preceding week**	
0	70.3 (539)
1–4	19.9 (153)
≥5	9.8 (75)

a Categories do not total 772 because of missing responses.

Approximately one-third of the sample had not walked for transportation in the previous week (34%), whereas 35% of the sample reported walking for transportation from 1 to 149 minutes in the previous week and 31% for more than 150 minutes in the previous week. Overall, 70% of participants reported they had not used public transportation in the previous week.

### Individual and public transportation correlates of walking for transportation

Table 2 summarizes the associations between personal characteristics and public transportation use with minutes spent walking for transportation in the previous week. The odds of meeting moderate physical activity guidelines by walking for transportation (OR, 2.08; CI, 1.27–3.42) and walking for transportation but not meeting the guidelines (OR, 2.11; CI, 1.31–3.40) were higher for participants who reported some public transportation use than for participants who did not use public transportation. Compared with participants who did not use public transportation, those who used public transportation for 5 or more days in the previous week were 8.61 times more likely to meet moderate physical activity guidelines by walking for transportation (CI, 3.87–19.20) and 3.47 times more likely to walk for transportation but not meet moderate physical activity guidelines ( CI, 1.47–8.19).

**Table 2:** Relationship Between Personal Characteristics and Public Transportation Use With Walking for Transportation Among Adults (N = 772) in St. Louis, Missouri, 2012

Personal Characteristic and Public Transportation Use	Walking for Transportation	
	1–149 Minutes/Week	≥150 Minutes/Week
	OR (95% CI)	OR (95% CI)
**Personal characteristic**		
**Sex**		
Male	1 [Reference]	1 [Reference]
Female	1.09 (0.75–1.57)	1.14 (0.77–1.68)
**Age, y**		
18–29	1 [Reference]	1 [Reference]
30–39	0.84 (0.47–1.50)	0.54 (0.30–0.97)
40–49	0.79 (0.43–1.45)	0.61 (0.33–1.13)
50–59	0.70 (0.38–1.31)	0.83 (0.46–1.52)
≥60	0.86 (0.47–1.58)	0.64 (0.34–1.19)
**Income, $**		
<10,000	1 [Reference]	1 [Reference]
10,000–29,999	1.15 (0.67–1.96)	1.10 (0.66–1.83)
30,000–49,999	1.13 (0.62–2.06)	0.65 (0.35–1.20)
50,000–69,999	1.38 (0.71–2.68)	0.51 (0.24–1.06)
≥70,000	1.60 (0.89–2.87)	0.70 (0.38–1.31)
**Employment status**		
Unemployed	1 [Reference]	1 [Reference]
Employed	0.95 (0.61–1.50)	1.16 (0.73–1.84)
**Public transportation use, days/previous week**		
0	1 [Reference]	1 [Reference]
1–4	2.11 (1.31–3.40)	2.08 (1.27–3.42)
≥5	3.47 (1.47–8.19)	8.61 (3.87–19.20)

Abbreviations: OR, odds ratio; CI, confidence interval

### Individual and perceived environmental correlates of public transportation use

Table 3 presents the associations between personal characteristics and perceived environmental factors with frequency categories of public transportation use in the previous week. The odds of using public transportation more than once a week (1–4 days/previous week) were significantly greater among participants aged 50 to 59 years (OR, 1.98; CI, 1.06–3.70) than among participants aged 18 to 29 years. Adults older than 60 were significantly less likely to have used public transportation 5 or more days in the previous week (OR, 0.34; CI, 0.14–0.86) than participants aged 18 to 29. Among other individual factors examined, employed participants were significantly less likely than unemployed participants to have used public transportation at least once in the previous week (1–4 days/previous wk) (OR, 0.56; CI, 0.35–0.92). This relationship differed for frequent public transportation use: the likelihood of having used public transportation 5 or more days in the previous week was significantly greater among employed participants than among the unemployed (OR, 2.99; CI, 1.52–5.90). For each income category examined, participants with higher incomes were significantly less likely to have used public transportation 1 to 4 days in the previous week than those with an annual income less than $10,000. Similarly, compared with participants earning less than $10,000 per year, participants with higher incomes were less likely to have used public transportation 5 or more days in the past week.

**Table 3:** Relationship of Personal Characteristics and Perceived Environmental Factors to Public Transportation Use Among Adults (N = 772) in St. Louis, Missouri

Personal Characteristics and Perceived Environmental Factors	Public Transportation Use	
	1–4 Days/Week	≥5 Days/Week
	OR (95% CI)	OR (95% CI)
**Personal characteristics**		
**Sex**		
Male	1 [Reference]	1 [Reference]
Female	1.21 (0.81–1.83)	1.23 (0.71–2.14)
**Age, y**		
18–29	1 [Reference]	1 [Reference]
30–39	1.00 (0.53–1.90)	0.95 (0.43–2.08)
40–49	1.34 (0.90–2.56)	0.93 (0.41–2.13)
50–59	1.98 (1.06–3.70)	1.69 (0.80–3.57)
≥60	0.86 (0.45–1.62)	0.34 (0.14–0.86)
**Income, $**		
<10,000	1 [Reference]	1 [Reference]
10,000–29,999	0.56 (0.35–0.92)	0.84 (0.45–1.57)
30,000–49,999	0.27 (0.13–0.51)	0.49 (0.23–1.03)
50,000–69,999	0.28 (0.13–0.58)	0.51 (0.21–1.20)
≥70,000	0.40 (0.24–0.70)	0.10 (0.03–0.34)
**Employment status**		
Unemployed	1 [Reference]	1 [Reference]
Employed	0.56 (0.35–0.92)	2.99 (1.52–5.90)
**Perceived environmental factors**		
**Speed of traffic**		
Disagree	1 [Reference]	1 [Reference]
Agree	0.54 (0.36–0.81)	0.76 (0.44–1.32)
**Perceived nearby crime**		
Disagree	1 [Reference]	1 [Reference]
Agree	1.06 (0.67–1.69)	0.50 (0.28–0.87)
**Safety from crime when walking during the day**		
Disagree	1 [Reference]	1 [Reference]
Agree	1.15 (0.73–1.82)	0.77 (0.41–1.43)
**Safety from crime when walking at night**		
Disagree	1 [Reference]	1 [Reference]
Agree	0.91 (0.53–1.58)	0.59 (0.29–1.18)
**Accessibility to sidewalks**		
Disagree	1 [Reference]	1 [Reference]
Agree	0.59 (0.30–1.16)	3.00 (0.69–13.12)
**Number of destinations within a 10-min walk**		
0	1 [Reference]	1 [Reference]
1–5	1.52 (0.72–3.23)	2.86 (0.78–10.49)
6–10	1.37 (0.61–3.10)	2.22 (0.57–8.60)
≥11	0.74 (0.30–1.81)	1.50 (0.36–6.30)

Abbreviations: OR, odds ratio; CI, confidence interval.

Among the perceived environmental factors assessed, the volume of traffic, presence of crosswalks and pedestrian signals, visibility of pedestrians and bicyclists, and street lighting at night were not significantly associated with public transportation use and were not included in the final, parsimonious model. Participants who reported that traffic exceeded the posted speed limits in their neighborhood were significantly less likely to have used public transportation for 1 to 4 days in the previous week (OR, 0.54; CI, 0.36–0.81) than those who did not report high traffic speeds in their neighborhood. Participants who perceived high crime in their neighborhood had significantly lower odds of having used public transportation for more than 5 days in the previous week (OR, 0.50; CI, 0.28–0.87) than those who did not report high crime. No significant associations were found for safety from crime when walking during the day, safety from crime when walking at night, accessibility of sidewalks, or accessibility of destinations.

## Discussion

Our study found that St. Louis adults who used public transportation were more likely to meet moderate physical activity recommendations by walking for transportation. Our findings are consistent with earlier research demonstrating that regular public transportation use is associated with increased physical activity (4–10,16,26). Therefore, public health efforts to support public transportation use may not only reduce traffic congestion, air pollution, and noise pollution in communities, but also may help people meet recommended levels of moderate physical activity (27).

Of the personal characteristics assessed in our study, age, employment, and income level were significantly associated with public transportation use. One key finding was that persons aged 60 years or older were less likely to use public transportation. As older adults often face mobility challenges, built environment features can accommodate this population, including bus shelters with seating, improved lighting, signage, and crosswalk signals that allow for slower walking speeds. The relationship between these types of built environment and transportation attributes with public transportation use among older adults is a topic for future investigation. Consistent with previous transportation research (28), our results showed that people with higher incomes were less likely to use public transportation. Because public transit may be the sole mode of transportation for lower-income people, future studies should examine the relationship in this population subgroup between perceptions of affordability and accessibility of public transportation with public transit use and with walking for transportation.

Our study builds on past research that has examined the relationship between perceptions of the built environment and walking for transportation (11–19) by assessing the specific, perceived environmental factors related to public transportation use. Our findings indicate that perceptions of high traffic speed and high crime were negatively associated with frequency of public transportation use. Previous transportation studies have examined the relationship between perceived and objective measures of crime nearby transit stops with bus and rail ridership and have suggested that the perception of high crime is a factor that can discourage public transit use (28–31). Loukaitou-Sideris and colleagues (29) found that crime at transit stops was higher where transit stops had poor environmental attributes, including graffiti, litter, or close proximity to undesirable establishments such as liquor stores or vacant buildings. In the same study, positive environmental characteristics, such as the presence of bus shelters and good visibility, were associated with lower crime at transit stops (29). These findings, along with those of our study, demonstrate the importance of future programs, policies, and infrastructure changes aimed at increasing public transportation use to improve perceived and actual safety from both crime and traffic. These types of investments, supportive of public transportation in St. Louis and in similar urban communities, could thereby increase walking for transportation.

Our study has several limitations. Our findings are based on cross-sectional data; thus, no causal relationships could be established. Although valid and reliable instruments were used, including the IPAQ and NEWS, data were collected via a self-administered questionnaire, and reporting biases may have occurred. Selection bias may have been introduced because of a low response rate; however, our response rate is similar to those of other studies on physical activity and transportation behaviors (15,32). In addition, we did not assess the intensity levels of the physical activity achieved by walking for transportation among this study sample. Finally, we did not explore the potential impact of certain transit service characteristics on public transportation use, including level of satisfaction with services associated with the public transportation system, type of public transportation service used, proximity to a public transportation stop, length of the public transportation trip, or the number of transfers taken on a typical trip. Future research should examine how these transit service characteristics may influence public transportation use and walking for transportation.

Despite these limitations, our results strengthen the evidence base demonstrating that public transportation use can support people in meeting moderate physical activity recommendations by walking for transportation (4–10,33). Our study also contributes to the limited body of existing literature regarding perceived environmental factors and public transportation use. We found that perceived environmental factors of traffic speed and neighborhood crime were negatively correlated with public transportation use in St. Louis. Public health and transportation professionals in St. Louis and elsewhere who want to encourage public transit use should implement strategies to decrease traffic speed on roadways through traffic calming, lowering speed limits, and increasing enforcement of traffic speed. Transportation planners and engineers should explore opportunities to enhance personal safety features on buses and trains and near transit stops to create safe environments for people using public transit. Public health and transportation professionals should also collaborate with law enforcement officials to increase police presence near bus and train stops. These changes have the potential to improve public transportation systems, which can provide more opportunities for people to meet recommended levels of physical activity and to achieve positive health outcomes.
